# Examining the relationship between food insecurity and causes of injury in Canadian adults and adolescents

**DOI:** 10.1186/s12889-021-11610-1

**Published:** 2021-08-17

**Authors:** Fei Men, Marcelo L. Urquia, Valerie Tarasuk

**Affiliations:** 1 Department of Nutritional Sciences, University of Toronto, Toronto, Ontario Canada; 2 Department of Consumer Sciences, The University of Alabama, Tuscaloosa, Alabama USA; 3 Department of Community Health Sciences, University of Manitoba, Winnipeg, Manitoba Canada; 4 Li Ka Shing Knowledge Institute, St Michael’s Hospital, Toronto, Ontario Canada

**Keywords:** Health inequity, Fall, Struck, Violence, Self-harm, Assault, Overexertion, Poisoning, Intentional injury, Unintentional injury

## Abstract

**Background:**

Food insecurity, as an indicator of socioeconomic disadvantages and a determinant of health, may be associated with injury by increasing risk exposure and hampering risk mitigation. We examined the association between food insecurity and common causes of injury in the general population.

**Methods:**

Linking the Canadian Community Health Survey 2005–2017 to National Ambulatory Care Reporting System 2003–2017, this retrospective cohort study estimated incidence of injury-related emergency department (ED) visits by food insecurity status among 212,300 individuals 12 years and above in the Canadian provinces of Ontario and Alberta, adjusting for prior ED visits, lifestyle, and sociodemographic characteristics including income.

**Results:**

Compared to those in food-secure households, individuals from moderately and severely food-insecure households had 1.16 (95% confidence interval [CI] 1.07–1.25) and 1.35 (95% CI 1.24–1.48) times higher incidence rate of ED visits due to injury, respectively, after confounders adjustment. The association was observed across sex and age groups. Severe food insecurity was associated with intentional injuries (adjusted rate ratio [aRR] 1.81; 95% CI 1.29–2.53) including self-harm (aRR 1.87; 95% CI 1.03–3.40) and violence (aRR 1.79; 95% CI 1.19–2.67) as well as non-intentional injuries (aRR 1.34; 95% CI 1.22–1.46) including fall (aRR 1.43; 95% CI 1.24–1.65), medical complication (aRR 1.39; 95% CI 1.06–1.82), being struck by objects (aRR 1.43; 95% CI 1.07–1.91), overexertion (aRR 1.31; 95% CI 1.04–1.66), animal bite or sting (aRR 1.60; 95% CI 1.08–2.36), skin piercing (aRR 1.80; 95% CI 1.21–2.66), and poisoning (aRR 1.65; 95% CI 1.05–2.59). Moderate food insecurity was associated with more injuries from violence (aRR 1.56; 95% CI 1.09–2.21), falls (aRR 1.22; 95% CI 1.08–1.37), being struck (aRR 1.20; 95% CI 1.01–1.43), and overexertion (aRR 1.25; 95% CI 1.04–1.50). Moderate and severe food insecurity were associated with falls on stairs and being struck in non-sports settings but not with falls on same level or being struck during sports. Food insecurity was not related to transport injuries.

**Conclusions:**

Health inequity by food insecurity status extends beyond diseases into differential risk of injury, warranting policy intervention. Researchers and policymakers need to address food insecurity as a social determinant of injury to improve health equity.

**Supplementary Information:**

The online version contains supplementary material available at 10.1186/s12889-021-11610-1.

## Background

Injury is the third leading cause of death in Canada, claiming over 17,000 lives in 2018, up from near 14,000 in 2005 [1]. Injury also led to over 2 million emergency department (ED) visits in 2017–18 [2], with direct and indirect costs projected to reach $75 billion by 2035 [3]. Falls were the most common injury in ED (31.6% of all injuries), followed by being struck by objects (14.1%) and transport incidents (7.9%) [2]. Intentional injuries accounted for 4.1% of all injury-related ED visits [2]. Compared to females, males were more likely to be injured by violence and transport incidents but less likely to self-harm [3]. Fall injuries mainly affected young children and seniors while youth were at higher risk of transport incidents and intentional injuries than people of other ages [2, 3].

Food insecurity, the inadequate or insecure access to food due to financial constraints, is experienced by one-eighth of Canadian households [4]. The ongoing COVID-19 pandemic may have exacerbated the problem [5, 6]. Food insecurity has been associated with chronic and infectious diseases [7–10], but little is known about its connection to non-disease outcomes such as injuries. However, three recent studies have associated food insecurity status with premature deaths from unintentional injuries and suicide among Canadian adults [10] and suicidal ideation among Canadian youth [11] and adults [12]. Canadian adults who had injury or illness among themselves or a relative or friend were over twice as likely to experience food insecurity than those who did not [13]. Child hunger – a sign of severe food insecurity – predicted fight injury and hospitalized injury among Canadian adolescents [14]. Among older adults in the US, being food-insecure was associated with self-reported falls [15]. Food insecurity may denote heightened risk of injury through increased exposure to hazardous environment and constrained ability to afford hazard mitigation [16]. In Canada, residents of deprived neighbourhoods are at elevated risk of being assaulted, falling, and drug poisoning [17–21]. This related to environmental hazards present in low-income neighbourhoods such as low social support, poor housing conditions, and high exposure to drugs and violence. Although Canadian studies of the neighbourhood-level correlates of food insecurity are lacking, the fact that food-insecure households are more likely to be low-income and rent rather than own their dwellings means they are also more likely to live in deprived neighbourhoods and thus be disproportionately exposed to these risks [22]. Exposure to hazardous work environments [23, 24] and household income inadequacy are also linked to both food insecurity and injuries [21, 22, 25–27]. Income is by definition a strong determinant of food insecurity [22], yet food insecurity is worthy of investigation in its own right given its strong and robust correlation with health above and beyond income [7, 9, 10]. For example, we found that food insecurity explained a far greater share of variance than income in the prevalence of chronic pain (16% vs 8%) and prescription opioids use (26% vs 4%) [8]. The relationship between food insecurity and injury may be bidirectional, with injury possibly aggravating food insecurity via worsened health and reduced employment capacity [28, 29]. Economic advantage may heighten the risk of certain types of injury by increasing exposure to injury-prone activities such as hockey and driving [14, 18, 25, 30, 31].

Food insecurity may be related to injury through the underlying socioeconomic disadvantages. The relevant research to date is scarce and limited to certain age groups [10, 11], self-reported injury measures [14, 15], and non-validated or binary measures of food insecurity [14, 15]. We use a validated scale of food insecurity severity and objective measures of injury to examine the relationship between food insecurity in a household and various causes of injury in a Canadian population sample. The universal health care system in Canada and its free-of-charge ED service minimize the cost-induced sample selection bias.

## Methods

### Population and sample

We linked Canadian Community Health Survey (CCHS) 2005–2017 to National Ambulatory Care Reporting System (NACRS) 2003–2017 to examine the association between food insecurity status and injury-related ED visits.

CCHS is an annual cross-sectional survey administered to roughly 65,000 households in Canada. One member 12 years or older is randomly selected per household to answer the survey. The responses generalize to 98% of the non-institutionalized population in the country. Questions on food insecurity have been formally incorporated in the survey since 2005 though certain jurisdictions chose not to answer them when given the option.

NACRS is the largest database of administrative records on ED visits in Canada, containing 64% of the ED cases nationwide. Ontario since 2002, Alberta since April 2010, and Yukon territory since 2015 have been mandated to report all ED records to NACRS; only a limited share of ED records were reported in other jurisdictions and years (e.g. 2% in Manitoba), where the reporting mandate was either partial or absent [2]. NACRS provides case-level information on timing and cause of each ED visit, which is assigned a main cause and up to nine joint causes, all coded in International Statistical Classification of Diseases and Related Health Problems, Tenth Revision, Canada (ICD-10-CA).

We limited our sample to Ontario and Alberta given their full coverage by NACRS. The two provinces combined represented roughly half of the country’s population. We linked all respondents from Ontario in CCHS 2005–2017 to NACRS 2003–2017 through unique person identifiers; likewise, respondents from Alberta interviewed in April/2012–2017 were linked to NACRS from April/2010–2017. Such linkages ensured taking full advantage of the available data to build the outcomes (injury-related ED visits in the past year) and a key covariate (ED visits in the year before). We excluded the other jurisdictions and years due to potential sampling bias; Yukon was excluded due to its lack of food insecurity measurement in CCHS 2005 and 2013–2016. Of the 241,500 survey respondents from Ontario and Alberta, we excluded 1800 individuals with invalid food insecurity status and all 27,400 Ontario respondents in CCHS 2015–2016 when the province opted out of food insecurity measurement. The final sample consisted of 212,300 individuals 12 years or older, of whom 20,900 had injury-related ED visits in the past year.

### Measures

Our primary outcome of interest was a count variable for the number of injury-related ED visits during the 12 months preceding the interview. We identified causes of injury mainly using the ICD-10-CA codes for secondary and tertiary causes of visit because main (primary) cause almost always described diagnosis (e.g. fracture) versus underlying causes (Table S1). As a sensitivity check, we constructed a narrower definition for injury, considering only the secondary cause. We separated intentional injuries from non-intentional injuries and examined two types of intentional injuries (violence, self-harm) and nine types of non-intentional injuries (fall, medical complications related to surgery and non-surgical interventions, being struck by objects, overexertion, transport incident, skin piercing, animal bite or sting, poisoning, and other miscellaneous). For transport incidents, medical complications, fall, and struck-by injuries, we further investigated subcategories under each. Given the demographic heterogeneity in injury risk [2, 3], we also stratified analyses of all-cause injury by sex and age.

Our key independent variable was food insecurity status in the past 12 months. This is a four-level variable built from the 18-item questionnaire in CCHS, developed by United States Department of Agriculture and adapted by Health Canada [32]. Ten questions asked about adults’ access to food in the past year while eight questions concerned food access among children below 18 years if there were any in the household. Based on the number of affirmative answers, a household was categorized as either food secure or marginally, moderately, or severely food insecure (Table S2).

We adjusted for factors that may confound the relationship between food insecurity and injury-related ED visits, including respondent’s sex (male, female), age at interview (years), race-ethnicity (white, Black, Indigenous, others), immigrant status (Canadian-born, immigrant), tobacco smoking status (never, former, current), past-year alcohol consumption (none, up to once a week, more than once a week), and number of ED visits in the year before (count, as a proxy for baseline health and injury risk). We also controlled for household characteristics including income categories (in $20,000 intervals), highest education (high school incomplete, high school graduate, some college, college degree), housing tenure (renter, homeowner), household type (couple with children, couple without children, lone parents, others), province (Ontario, Alberta), and survey cycle. We selected the confounding variables a priori based on their established associations with injury [10, 14, 15, 21, 24, 26, 28, 30, 31]. Missing values in covariates were dummy coded and kept in the analyses.

### Statistical analyses

We used chi-squared tests and t-tests to compare sample characteristics by injury status; we also used t-tests and trends analyses to compare crude rates of injuries across food insecurity levels. We fitted Poisson models on the all-cause and cause-specific injury outcomes, adjusting for confounding factors. Models on all-cause injury were further stratified by sex and age. We conducted sensitivity analyses on all-cause injury, expanding the sample to include jurisdiction-years with partial ED records, using a narrower definition of injury, and applying person weights, respectively. Given that most people from our sample did not visit ED for injury in the past year, we also experimented with standard and zero-inflated negative binomial models, respectively, to compare their fitness to data to the Poisson model’s. We showed two-sided robust confidence intervals. Results were considered statistically significant at *p* < 0.05. Analyses were conducted unweighted in Stata SE 15.1. Numbers of observations were rounded to protect respondents’ identity. Ethics approval was obtained from the Health Sciences Research Ethics Board at the University of Toronto.

## Results

Our sample contained 212,300 respondents 12 years and older in Ontario and Alberta, Canada (Table 1). A total of 3.6, 4.9, and 2.5% of the sample lived in marginally, moderately, and severely food-insecure households, respectively. Compared to respondents without past-year injury-related ED visits, injury-related ED visitors were more likely to be female, older, and from socioeconomically disadvantaged households (e.g. lower income, less education). Over half of the visitors and near one-third of the non-visitors used an ED in the year before past year.

**Table 1:** Sample characteristics by past-year injury-related ED visit status among respondents of CCHS 2005–2017

	No injury-related ED visit	Any injury-related ED visit	Total
Household food insecurity			
Food-secure	89.4	85.4	89.0
Marginal food insecurity	3.5	4.1	3.6
Moderate food insecurity	4.7	6.3	4.9
Severe food insecurity	2.3	4.2	2.5
Sex			
Male	45.0	50.1	45.5
Female	55.0	49.9	54.5
Age (years) ± SD	48.4 ± 20.6	45.5 ± 22.3	48.1 ± 20.8
Race-ethnicity			
White	84.8	87.7	85.1
Black	1.7	1.0	1.6
Indigenous	9.5	5.2	9.0
Others	3.2	5.1	3.4
Not stated	0.8	1.0	0.9
Immigrant status			
Canadian-born	80.1	85.8	80.6
Immigrant	19.7	14.1	19.1
Not stated	0.3	0.2	0.3
Household income (Canadian dollar)			
Less than $20,000	7.0	8.8	7.2
$20,000 - 39,999	14.6	15.4	14.7
$40,000 - 59,999	13.9	13.6	13.9
$60,000 - 79,999	12.3	11.5	12.2
$80,000 or more	30.6	27.9	30.4
Not stated	21.5	22.7	21.6
Highest education in household			
High school incomplete	13.3	13.9	13.4
High school graduate	9.0	10.2	9.2
Some college	3.5	3.9	3.5
College degree	69.8	66.8	69.5
Not stated	4.3	5.2	4.4
Housing tenure			
Renter	21.3	25.1	21.7
Homeowner	78.5	74.7	78.2
Not stated	0.1	0.2	0.1
Household type			
Couples with children	32.7	33.8	32.9
Couples without children	31.5	27.2	31.1
Lone parents	8.0	10.2	8.2
Others	27.4	28.4	27.5
Not stated	0.3	0.4	0.3
Province of residence			
Ontario	85.9	84.0	85.7
Alberta	14.1	16.0	14.3
Tobacco smoking status			
Never smoked	40.7	38.7	40.5
Former smoker	40.1	37.1	39.8
Current smoker	19.1	24.0	19.6
Not stated	0.1	0.2	0.1
Past-year alcohol consumption			
None	46.9	47.2	46.9
Any up to once a week	23.7	25.5	23.8
More than once a week	29.2	26.9	28.9
Not stated	0.3	0.4	0.3
CCHS cycle			
Cycle 2005–2006	15.9	15.8	15.9
Cycle 2007–2008	16.7	16.3	16.7
Cycle 2009–2010	15.8	15.4	15.8
Cycle 2011–2012	16.9	16.3	16.8
Cycle 2013–2014	19.7	19.8	19.7
Cycle 2015–2016	5.3	5.7	5.3
Cycle 2017	9.7	10.6	9.8
ED visit 13–24 months ago			
No	67.8	48.0	65.9
Yes	32.2	52.0	34.1
Frequency in a year (times)	0.91 ± 4.23	2.13 ± 8.50	1.03 ± 4.83
*Number of respondents*	*191,400*	*20,900*	*212,300*

Notes: ED = emergency department. All differences between any injury and no injury are significant at *p* < 0.001 based on chi-square tests for the categorical variables and t-tests for age and frequency of ED visits 13–24 months ago

For every 10,000 persons traced in the past year, there were 11,090 all-cause ED visits, of which 11.6% (1286) were injury-related (Table 2). The incidence rate of intentional injuries was 27 per 10,000 person-years, with over twice as many incidents of violence (19) as self-harm (8). Non-intentional injuries were much more common, with 967 incidents per 10,000 person-years. Falls (315), medical complications (114), and being struck by objects (112) were the most common non-intentional injuries, followed by overexertion (103) and transport-related injuries (90). An estimated 9.4% of food-secure people and 11.3, 12.7, and 16.2% of marginally, moderately, and severely food-insecure people respectively visited an ED due to injury in the past year. As food insecurity worsened, ED visits became more frequent for all causes of injury except being struck during sports, poisoning, and non-surgical medical complications (trends *p* > 0.05). Injury-related ED visits were also more common among food-insecure versus food-secure people for men, women, non-senior adults 18–64 years old, and seniors 65 years and older, but not adolescents 12–17 years old (trends *p* > 0.05; Table S3).

**Table 2:** Rate per 10,000 person-years of past-year ED visits in the overall sample

	Food-secure	Marginal FI	Moderate FI	Severe FI	Total
All-cause ED visit	10,526	12830*	15340*	20252*	11,090
Injury-related ED visits	1224	1458*	1760*	2291*	1286
Intentional injuries	20	55*	79*	131*	27
Self-harm	6	18	22*	37*	8
Violence	14	37*	57*	93*	19
Non-intentional injuries	930	55*	79*	131*	967
Fall	302	338	409*	532*	315
On same level	136	158	169*	194*	140
On stairs	39	47	73*	104*	43
Others	128	133	167*	233*	132
Medical complication	110	121	141	190*	114
Surgical	92	88	117	151*	95
Non-surgical	64	55	90	95	66
Struck-by	106	139*	162*	183*	112
Falling objects	19	26	29	32	20
In sports	41	47	43	32	41
In non-sports	52	78*	103*	160*	59
Transport	85	116*	125*	175*	90
Pedestrian or cyclist	19	24	35	35*	20
Motor vehicle	63	87	82	136*	67
Overexertion	98	112	153*	177*	103
Animal bite or sting	42	53	52	80*	44
Skin piercing	27	32	30	58*	28
Poisoning	26	25	45	48	27
Other non-intentional injuries	373	444*	501*	655*	389
*Number of respondents*	*188,900*	*7600*	*10,400*	*5400*	*212,300*

Notes: ED = emergency department. FI = food insecurity. Non-intentional injuries included unintentional injuries and a negligible share of injuries with “undetermined intent”. All rates of the food-insecure categories were significantly different from those of the food-secure at *p *< 0.05 are denoted with asterisk *. All trends were significant at p < 0.05 except “being struck in sports” with *p* = 0.77, “poisoning” with *p* = 0.067, and “non-surgical medical complications” with *p* = 0.15. “Non-motor transport” had *p* = 0.057 for moderate FI vs *p* = 0.039 for severe FI; the rate was 35 per 10,000 person-years for both after rounding. Rates from subcategories do not necessarily sum to the exact rates from their corresponding categories due to rounding

Marginal, moderate, and severe food insecurity were associated with 1.19 (95% confidence interval [CI] 1.10–1.29), 1.44 (95% CI 1.34–1.54), and 1.87 (95% CI 1.72–2.04) times higher incidence rate of injury-related ED visits in the past year, respectively (Table 3). The incidence rate ratios became smaller after confounders adjustment, leaving marginal food insecurity non-significant; moderate and severe food insecurity were still associated with 1.16 (95% CI 1.07–1.25) and 1.35 (95% CI 1.24–1.48) times higher incidence rate of injury, respectively (See Table S4 for all covariates’ coefficients; Table S5 for *p-value* of the food insecurity variable; Fig. S1 for predicted probabilities). Expanding the sample, narrowing the definition of injury, applying weights, or fitting standard or zero-inflted negative binomial models did not change the result (Table S6). Moderate food insecurity was associated with higher incidence of injury among women (adjusted rate ratio [aRR] 1.23, 95% CI 1.10–1.37) but not men (aRR 1.03, 95% CI 0.93–1.14; sex interaction *p* = 0.011); severe food insecurity was equally associated with injury for both sexes (aRR 1.37 for men and 1.38 for women, *p* < 0.05 for both). Moderate food insecurity predicted higher incidence rate of injury similarly across ages while severe food insecurity was associated with injury among adolescents (aRR 1.25, 95% CI 1.001–1.57) and non-senior adults (aRR 1.43, 95% CI 1.29–1.58; greater than aRR for adolescents and seniors, age interactions *p *< 0.05) but not among seniors.

**Table 3:** Incidence rate ratio of injury-related ED visits by food insecurity status, in the overall sample and subsamples by sex and age

	Food-secure	Marginal FI	Moderate FI	Severe FI
Injury-related ED visit, unadjusted (*n* = 212,300)	reference	1.19 (1.10–1.29)	1.44 (1.34–1.54)	1.87 (1.72–2.04)
Injury-related ED visit (n = 212,300)	reference	1.02 (0.93–1.11)	1.16 (1.07–1.25)	1.35 (1.24–1.48)
Male (*n* = 96,700)	reference	1.00 (0.88–1.13)	1.03 (0.93–1.14)	1.37 (1.19–1.59)
Female (*n* = 115,600)	reference	1.06 (0.95–1.18)	1.23 (1.10–1.37)	1.38 (1.24–1.54)
12–17 years old (*n* = 18,600)	reference	0.96 (0.80–1.15)	1.19 (1.03–1.39)	1.25 (1.00–1.57)
18–64 years old (*n* = 140,200)	reference	1.07 (0.98–1.17)	1.16 (1.07–1.27)	1.43 (1.29–1.58)
65+ years old (*n* = 53,500)	reference	0.89 (0.60–1.32)	1.29 (1.01–1.65)	1.17 (0.81–1.71)

Notes: ED = emergency department. FI = food insecurity. All estimates are from Poisson models; 95% confidence intervals are shown in parentheses after incidence rate ratios. Unless specified otherwise, all Poisson models adjusted for sex, age, race-ethnicity, immigrant status, household income, income imputation status, highest education in household, housing tenure, household type, jurisdiction of residence, smoking status, past-year alcohol consumption, CCHS cycle, and number of ED visits in the year before

**Fig. 1:**
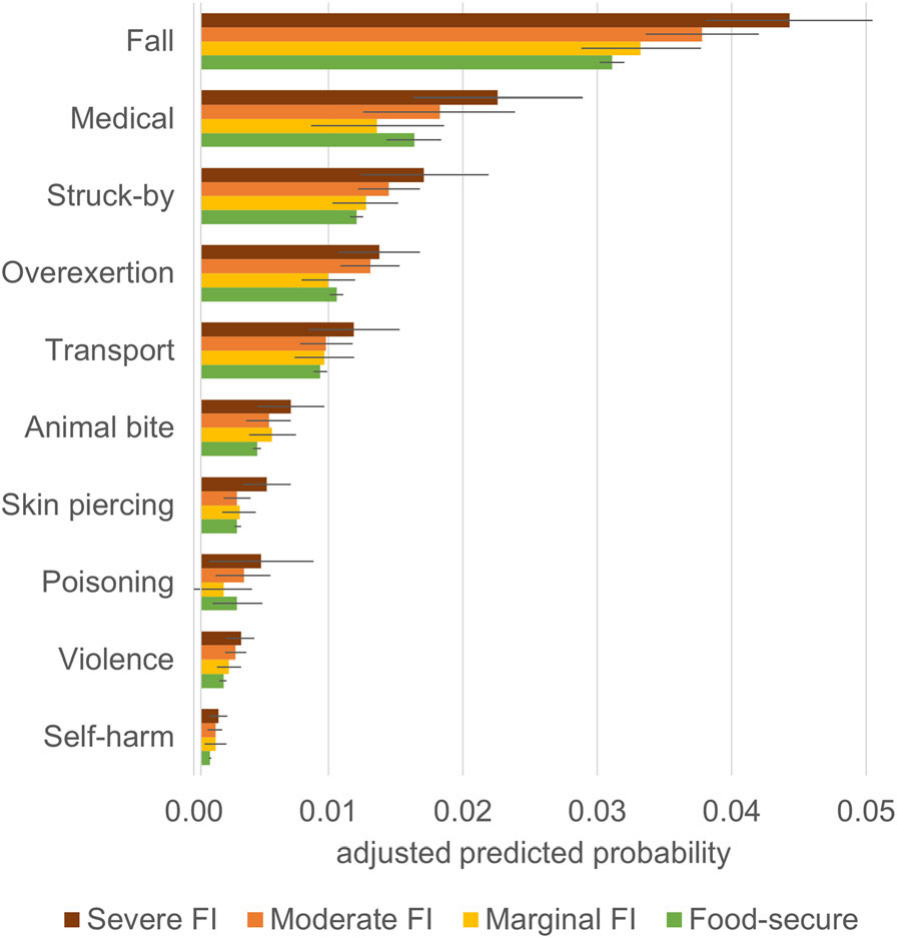
Adjusted predicted probability of cause-specific injury by food insecurity status: overall sample. Black horizontal lines represented 95% confidence intervals. Average probabilities were estimated through marginal standardization based on the adjusted Poisson models

After adjusting for confounders, moderate and severe food insecurity were associated with 1.54 (95% CI 1.14–2.10) and 1.81 (95% CI 1.29–2.53) times higher incidence rate of intentional injuries, respectively; the comparable figures for non-intentional injuries were 1.14 (95% CI 1.06–1.24) and 1.34 (95% CI 1.22–1.46) (Table 4; Table S7 for *p-value* of the food insecurity variable). As with intentional injuries, moderate and severe food insecurity were associated with 1.56 (95% CI 1.09–2.21) and 1.79 (95% CI 1.19–2.67) times higher adjusted incidence rate of victimization by violence, respectively. Severe food insecurity was further associated with 1.87 (95% CI 1.03–3.40) times greater adjusted incidence rate of self-harm. As with non-intentional injuries, severe food insecurity was associated with higher likelihood of falling (aRR 1.43, 95% CI 1.24–1.65), medical complications (aRR 1.39, 95% CI 1.06–1.82), being struck by objects (aRR 1.43, 95% CI 1.07–1.91), overexertion (aRR 1.31, 95% CI 1.04–1.66), animal bite or sting (aRR 1.60, 95% CI 1.08–2.36), skin piercing (aRR 1.80, 95% CI 1.21–2.66), accidental poisoning (aRR 1.65, 95% CI 1.05–2.59), and other miscellaneous non-intentional injuries (aRR 1.32, 95% CI 1.15–1.52). Moderate food insecurity was further associated with higher incidence rate of fall (aRR 1.22, 95% CI 1.08–1.37), being struck (aRR 1.20, 95% CI 1.01–1.43), and overexertion (aRR 1.25, 95% CI 1.04–1.50) (Fig. 1 for predicted probability).

**Table 4:** Incidece rate ratio of injury-related ED visits due to cause-specific injury by food insecurity status in overall sample

	Food-secure	Marginal FI	Moderate FI	Severe FI
**Unadjusted**				
Intentional injuries	reference	2.76 (1.84–4.14)	3.95 (2.98–5.22)	6.53 (4.86–8.76)
Self-harm	reference	3.05 (1.45–6.41)	3.67 (2.19–6.15)	6.18 (3.61–10.60)
Violence	reference	2.63 (1.70–4.09)	4.07 (2.92–5.65)	6.67 (4.69–9.49)
Non-intentional injuries	reference	1.16 (1.08–1.26)	1.40 (1.30–1.50)	1.79 (1.65–1.95)
Fall	reference	1.12 (0.97–1.28)	1.35 (1.21–1.51)	1.76 (1.53–2.02)
On same level	reference	1.16 (0.95–1.42)	1.25 (1.06–1.47)	1.43 (1.15–1.77)
On stairs	reference	1.21 (0.84–1.75)	1.88 (1.45–2.42)	2.68 (1.98–3.62)
Others	reference	1.04 (0.84–1.28)	1.30 (1.10–1.55)	1.83 (1.49–2.24)
Medical complication	reference	0.92 (0.71–1.18)	1.32 (0.96–1.81)	1.57 (1.22–2.03)
Surgical	reference	0.95 (0.70–1.31)	1.26 (0.90–1.76)	1.63 (1.23–2.17)
Non-surgical	reference	0.86 (0.57–1.31)	1.40 (0.78–2.51)	1.49 (0.94–2.34)
Struck-by	reference	1.35 (1.10–1.65)	1.56 (1.33–1.83)	1.99 (1.47–2.71)
Falling objects	reference	1.39 (0.87–2.23)	1.53 (1.03–2.27)	1.68 (1.00–2.80)
In sports	reference	1.15 (0.80–1.67)	1.06 (0.76–1.46)	0.77 (0.47–1.28)
In non-sports	reference	1.48 (1.12–1.96)	1.97 (1.59–2.43)	3.06 (2.05–4.57)
Transport	reference	1.36 (1.07–1.73)	1.47 (1.19–1.83)	2.07 (1.55–2.75)
Pedestrian or cyclist	reference	1.28 (0.77–2.11)	1.87 (1.15–3.05)	1.91 (1.20–3.04)
Motor vehicle	reference	1.37 (1.03–1.82)	1.29 (1.01–1.65)	2.15 (1.52–3.04)
Overexertion	reference	1.14 (0.92–1.42)	1.56 (1.32–1.85)	1.81 (1.45–2.25)
Animal bite or sting	reference	1.26 (0.90–1.77)	1.25 (0.91–1.71)	1.92 (1.37–2.70)
Skin piercing	reference	1.18 (0.75–1.85)	1.11 (0.77–1.60)	2.15 (1.50–3.10)
Poisoning	reference	0.96 (0.44–2.08)	1.74 (0.88–3.43)	1.87 (0.90–3.87)
Other non-intentional injuries	reference	1.19 (1.04–1.36)	1.34 (1.19–1.52)	1.76 (1.55–2.00)
**Adjusted**				
Intentional injuries	reference	1.37 (0.91–2.05)	1.54 (1.14–2.10)	1.81 (1.29–2.53)
Self-harm	reference	1.61 (0.77–3.34)	1.51 (0.84–2.72)	1.87 (1.03–3.40)
Violence	reference	1.27 (0.81–2.00)	1.56 (1.09–2.21)	1.79 (1.19–2.67)
Non-intentional injuries	reference	1.01 (0.92–1.10)	1.14 (1.06–1.24)	1.34 (1.22–1.46)
Fall	reference	1.07 (0.93–1.23)	1.22 (1.08–1.37)	1.43 (1.24–1.65)
On same level	reference	1.10 (0.90–1.35)	1.11 (0.94–1.32)	1.15 (0.92–1.45)
On stairs	reference	1.16 (0.80–1.68)	1.71 (1.30–2.26)	2.24 (1.62–3.10)
Others	reference	1.00 (0.81–1.24)	1.18 (0.98–1.42)	1.49 (1.20–1.85)
Medical complication	reference	0.83 (0.54–1.27)	1.12 (0.77–1.61)	1.39 (1.06–1.82)
Surgical	reference	0.84 (0.53–1.33)	1.05 (0.70–1.59)	1.32 (0.94–1.85)
Non-surgical	reference	0.83 (0.44–1.55)	1.17 (0.61–2.27)	1.49 (0.99–2.25)
Struck-by	reference	1.06 (0.86–1.30)	1.20 (1.01–1.43)	1.43 (1.07–1.91)
Falling objects	reference	1.15 (0.72–1.84)	1.20 (0.78–1.83)	1.19 (0.69–2.04)
In sports	reference	0.97 (0.67–1.41)	1.02 (0.73–1.43)	0.89 (0.52–1.52)
In non-sports	reference	1.14 (0.86–1.52)	1.40 (1.11–1.75)	1.83 (1.26–2.66)
Transport	reference	1.03 (0.81–1.32)	1.05 (0.85–1.31)	1.29 (0.94–1.75)
Pedestrian or cyclist	reference	0.87 (0.52–1.45)	1.15 (0.73–1.83)	1.00 (0.58–1.74)
Motor vehicle	reference	1.06 (0.80–1.42)	0.95 (0.73–1.23)	1.38 (0.96–1.99)
Overexertion	reference	0.94 (0.75–1.17)	1.25 (1.04–1.50)	1.31 (1.04–1.66)
Animal bite or sting	reference	1.27 (0.90–1.80)	1.20 (0.86–1.68)	1.60 (1.08–2.36)
Skin piercing	reference	1.05 (0.67–1.65)	0.99 (0.68–1.45)	1.80 (1.21–2.66)
Poisoning	reference	0.62 (0.10–3.88)	1.16 (0.35–3.86)	1.65 (1.05–2.59)
Other non-intentional injuries	reference	1.00 (0.87–1.15)	1.09 (0.96–1.23)	1.32 (1.15–1.52)

Notes: ED = emergency department. FI = food insecurity. The “adjusted” Poisson models controlled for sex, age, race-ethnicity, immigrant status, household income, income imputation status, highest education in household, housing tenure, household type, jurisdiction of residence, smoking status, past-year alcohol consumption, CCHS cycle, and number of ED visits in the year before

There were further nuances with some non-intentional injuries (Fig. S2 for predicted probability). The adjusted associations with falls were primarily driven by falls on stairs (moderate food insecurity aRR 1.71, 95% CI 1.30–2.26; severe food insecurity aRR 2.24, 95% CI 1.62–3.10) and other miscellaneous falls (severe food insecurity aRR 1.49, 95% CI 1.20–1.85). The significant results with being struck were mainly driven by non-sports cases where moderate and severe food insecurity were associated with 1.40 (95% CI 1.11–1.75) and 1.83 (95% CI 1.26–2.66) times higher incidence rate than food security. Injuries from transport incidents, surgical or non-surgical medical complications, falls on same level, and being struck by falling objects or in sports settings were unrelated to food insecurity in the adjusted models.

## Discussion

Using 14 years of Canadian ED records, we found moderate and severe food insecurity associated with higher incidence rate of injury. The association was found across sex and age subsamples. Compared to individuals who were food-secure, severely food-insecure people appeared more susceptible to injuries except those related to transport; moderately food-insecure people were prone to injuries by violence, falls, being struck by objects, and overexertion. The results by and large conformed with previous literature associating food hardships with injury [10, 11, 14, 15, 28]. We showed that food insecurity, as a proxy for socioeconomic disadvantages, is associated with injury above and beyond income and other common socioeconomic indicators.

While identifying the precise mechanism connecting food insecurity to injury is beyond the scope of this paper, our findings accorded with our understanding of the association between socioeconomic disadvantages and injury [16]. That food insecurity correlated with violence may reflect exposure of food-insecure individuals to less safe environments relative to their food-secure counterparts, whether at home [20], school [14], workplace [28], or neighborhood [17]. Violence is more likely to occur in neighborhoods with high levels of perceived social disorders such as prevalence of abandoned houses and high incidence of assaults, possibly due to the weakening of trust and connections among neighbors [20]. Food-insecure people may be disproportionately affected given that their resource constraints limit their choice of neighbourhoods, and that they are more likely to report a weak sense of community belonging [33]. The feelings of shame and powerlessness associated with food insecurity could further magnify food-insecure individuals’ vulnerability to assaults insofar as they appear as easy targets [9]. Moreover, exposure to intimate partner violence may affect victims’ ability to afford food, especially for women [28, 29, 34], which could help explain the sex differential we found. The connection between severe food insecurity and self-harm aligns with prior findings on suicidality [10–12]. Chronic stress, mental disorders, substance use, and social isolation are all strong correlates of food insecurity potentially raising the risk of self-harm [7–9, 11, 12, 33, 35]. The significant association with being struck in non-sports versus sports settings suggests that food-insecure individuals may work and live in environments with more struck-by injury hazards (e.g. construction work) but participate less in risky and costly sports (e.g. hockey) compared to their food-secure counterparts [14, 23]. Given the high prevalence of sports-related injury among youth [2], the null relationship between food insecurity and adolescents’ injury risk is unsurprising. Overexertion and skin piercing are common occupational injuries in manual labor with strenuous work and contact with sharp objects [24, 26] whereas bites and stings are caused by animal aggression. Food-insecure people may have greater exposure to these risks than their food-secure counterparts due to exposure to more hazardous neighborhoods and workplace environments [19, 23].

Health problems (e.g. cardiovascular diseases) and poor housing conditions (e.g. inadequate lighting, slippery floor, housing in need of major repairs), which are key determinants of fall injuries, disproportionately affect the food-insecure population [7, 36]. Compared to food-insecure people, the food-secure may be better able to maintain their dwellings and afford measures such as shock-absorbing flooring and prescription medications that mitigate the risk of fall injuries [27]. That food insecurity correlated with falls on stairs but not falls on same level could be because the latter encompassed falls during recreational activities (e.g. ice-skating) disproportionately practiced by food-secure people.

People from food-insecure households are more likely to use prescription opioids for pain management than their food-secure counterparts [8], which could potentially lead to differential exposure to poisoning risk. Moreover, higher-income opioid users are more likely to afford antagonists than their lower-income counterparts [37], justifying the food-insecure people’s higher susceptibility to opioid poisoning. Likewise, food-insecure adults are more likely to have surgeries [7] but less likely to receive post-surgery rehabilitation [38], heightening their risk of medical complications. While prior research has found economic disadvantages both positively and negatively associated with transport-related injuries [18, 21, 39], we found no such connection with food insecurity status, possibly due to the increasing traffic safety and more equitable access to transport means over time [40, 41].

This study advances our understanding of food insecurity as a social determinant of health, not just for mental or metabolic health but also for vulnerability to injury, an aspect largely overlooked to date. The findings are consistent with our knowledge of the serious and pervasive material deprivation that characterizes moderate and severe food insecurity, which is related to - yet fundamentally different from - other measures of socioeconomic disadvantages such as low income [8, 42]. Unlike income, the household-level measure of food insecurity used in this study is an experience-based measure of people’s ability to access adequate food, which is arguably a more sensitive and specific indicator of material hardship. While low income is a common cause of food insecurity, income itself cannot capture the negative impact of low assets, high living cost, and large household size on material wellbeing as food insecurity does. This study reinforces the importance of policy interventions to reduce food insecurity [43–46]. Measures targeting at-risk populations could potentially reduce socioeconomic disparity in injury. Financial assistance for home adaptation among low-income individuals with mobility issues may lower their financial barriers to improving housing condition and reducing their risk of fall injury [47, 48]. Certain safety-for-all measures may also reduce socioeconomic disparity in injury risk given the disproportionately high share of food-insecure individuals at risk. For instance, universal insurance coverage on prescription medications, mandatory training on use of protective equipment for construction workers, and free distribution of opioid antagonist affect all intended targets irrespective of their food insecurity status, but the effect of injury reduction may be particularly pronounced among those experiencing food insecurity given their higher likelihood in prescription nonadherence, fall injury, and opioid overdose [8, 23, 27].

### Limitations

The use of a multi-year population sample, a validated food insecurity scale, and objectively measured causes of injury were our strengths. Caveats are also worth noting. Directionality is not established in our findings. Food insecurity could be a distant driver of injury while injury - through its impact on income, mobility, and mental health - could increase the risk of food insecurity as well. Unobserved factors may also cause food insecurity and injury simultaneously. Assuming food insecurity as a distal cause of injury, there are mechanisms leading to different types of injury, which go beyond the scope of this paper and need separate analysis. Social desirability bias may lead some patients to misreport injury causes due to shame (e.g. domestic violence). ED health professionals may also avoid coding injuries as intentional due to legal implications (e.g. mandatory reporting of sexual assaults) [17]. Such biases tend to yield undercounting of intentional injuries and overcounting of unintentional ones though the magnitude of misclassification and its impact on our results remain undetermined. The sample was restricted to Ontario and Alberta, limiting the generalizability of our findings to these two provinces. Injuries that result in ED visits are usually more serious and may resemble fatal injuries in their relationship with socioeconomic disadvantages [16]. Whether injuries not treated in ED differ by food insecurity status is left to future investigation. Our sample was predominantly white. The very low number of non-white participants precluded the stratification of analyses by race-ethnicity. While this does not affect the validity of our findings, the moderating effect of race-ethnicity on the association between food insecurity and injury warrants further studies.

## Conclusions

Food insecurity is significantly associated with injury-related ED visits in the Canadian population. Policies ought to address food insecurity as a social determinant of injury to improve health equity.

## Supplementary Information

**Additional file 1: Table S1.** ICD-10-CA code for injury-related ED visits. **Table S2.** Food insecurity status, based on CCHS 18-item questionnaire. **Table S3.** Rate per 10,000 persons of past-year injury-related ED visit by food insecurity status, stratified by sex and age. **Table S4.** Incidence rate ratio from adjusted Poisson model on past-year injury-related ED visits in overall sample. **Table S5.** Incidence rate ratio from Poisson models on past-year injury-related ED visits in overall sample and by sex and age subsamples. **Table S6.** Sensitivity test on all-cause injury-related ED visits. **Table S7.** Poisson models on past-year ED visits due to cause-specific injuries in overall sample . **Fig. S1.** Adjusted predicted probability of injury by food insecurity status: overall sample and by sex and age subsamples. **Fig. S2.** Adjusted predicted probability of specific non-intentional injury by food insecurity status: overall sample.

## Data Availability

The data that support the findings of this study are available from Statistics Canada Research Data Centre but restrictions apply to the availability of these data, which were used under license for the current study, and so are not publicly available. Data are however available from the authors upon reasonable request and with permission of Statistics Canada Research Data Centre.
