# Supplementary material for: Examining the relationship between food insecurity and causes of injury in Canadian adults and adolescents
Source: BMC Public Health. 2021 Aug 17;21:1557. doi: 10.1186/s12889-021-11610-1 (PMC8367649; doi:10.1186/s12889-021-11610-1)
Supplement: Supplementary file 1 — Additional file 1: Table S1. ICD-10-CA code for injury-related ED visits. Table S2. Food insecurity status, based on CCHS 18-item questionnaire. Table S3. Rate per 10,000 persons of past-year injury-related ED visit by food insecurity status, stratified by sex and age. Table S4. Incidence rate ratio from adjusted Poisson model on past-year injury-related ED visits in overall sample. Table S5. Incidence rate ratio from Poisson models on past-year injury-related ED visits in overall sample and by sex and age subsamples. Table S6. Sensitivity test on all-cause injury-related ED visits. Table S7. Poisson models on past-year ED visits due to cause-specific injuries in overall sample . Fig. S1. Adjusted predicted probability of injury by food insecurity status: overall sample and by sex and age subsamples. Fig. S2. Adjusted predicted probability of specific non-intentional injury by food insecurity status: overall sample. [file 12889_2021_11610_MOESM1_ESM.docx]

**Supplement**

| Table S1. ICD-10-CA code for injury-related ED visits | |  |
| --- | --- | --- |
| Intentional injuries | X60-Y09, Y870, Y871, T741, T742, Z044, Z0450, Z0451, Z614, Z615 |  |
| Self-harm | X60-X84, Y870 |  |
| Violence | X85-Y09, Y871, T741, T742, Z044, Z0450, Z0451, Z614, Z615 |  |
| Non-intentional injuries | V, W, X01-X59, Y10-Y86, Y872, Y88, Y89 |  |
| Fall | W00-W19 |  |
| On same level | W00-W03 |  |
| On stairs and steps | W10 |  |
| Others | W04-W09, W11-W19 |  |
| Medical complication | Y40-84 |  |
| Surgical | Y83 |  |
| Non-surgical | Y40-Y82, Y84 |  |
| Struck-by | W20-22 |  |
| Thrown, projected, falling objects | W20 |  |
| In sports | W21, W2200-W2207 |  |
| In non-sports or unspecified | W2208-W2209 |  |
| Transport | V, Y850, Y859 |  |
| Pedestrian or cyclist | V01-19, Y859 |  |
| Motor vehicle or unspecified | V20-V99, Y850 |  |
| Overexertion | X50 |  |
| Animal bite or sting | W53-W59, X23 |  |
| Skin piercing | W45 |  |
| Poisoning | X40-X49, Y450, Y578, Y579 |  |
| Other non-intentional injuries | Others not listed above in V, W, X01-X59, Y10-Y86, Y872, Y88, Y89 |  |
| Injury | Any of above as primary, secondary, or tertiary cause of ED visit |  |
| Alternative injury | V, W, X, Y01-Y89 as secondary cause of ED visit |  |
| Notes: Injuries denoted by V, W, X, and Y are identified through secondary or tertiary causes ("other_problem_1" and "other_problem_2" in NACRS). Violence also included injuries of T and Z classes as main ("main_problem" in NACRS), secondary, or tertiary cause. | |  |
|  |  |  |
|  |  |  |

| Table S2. Food insecurity level, based on CCHS 18-item questionnaire | | |
| --- | --- | --- |
| Status | Measurement | Interpretation |
| Food-secure | Affirmed no item on either the 10-item adult food security scale or 8-item child food security scale | No report of income-related problems of food access. |
| Marginally food-insecure | Affirmed no more than 1 item on either scale | Some indication of worry or an income-related barrier to adequate, secure food access. |
| Moderately food-insecure | Affirmed 2 to 5 items on the adult scale or 2 to 4 items on the child scale | Compromise in quality and/or quantity of food consumed by adults and/or children due to a lack of money for food. |
| Severely food-insecure | Affirmed more than 5 items on the adult scale or more than 4 items on the child scale | Disrupted eating patterns and reduced food intake among adults and/or children due to a lack of money for food |
|  | | |

| Table S3. Rate per 10,000 persons of past-year injury-related ED visit by food insecurity status, stratified by sex and age | | | | | |  |
| --- | --- | --- | --- | --- | --- | --- |
|  | Food-secure | Marginal FI | Moderate FI | Severe FI | Total |  |
| Male (n=96,700) | 1365 | 1641* | 1751* | 2579* | 1417 |  |
| Female (n=115,600) | 1104 | 1321* | 1766* | 2106* | 1176 |  |
| 12-17 years old (n=18,600) | 1896 | 1744 | 2142 | 2563* | 1924 |  |
| 18-64 years old (n=140,200) | 1126 | 1413* | 1681* | 2320* | 1206 |  |
| 65+years old (n=53,500) | 1257 | 1393 | 1821* | 1514 | 1274 |  |
| Notes: ED = emergency department. FI = food insecurity. All rates significantly different from those of the food-secure at p<0.05 are denoted with asterisk *. All trends analyses on food insecurity status are all significant at p<0.05 except for 12-17 years old. | | | | | |  |
|  |  |  |  |  |  |  |

| Table S4. Incidence rate ratio from adjusted Poisson model on past-year injury-related ED visits in overall sample | |  |
| --- | --- | --- |
| Household food insecurity |  |  |
| Food-secure (reference) | 1.00 (1.00-1.00) |  |
| Marginal food insecurity | 1.02 (0.93-1.11) |  |
| Moderate food insecurity | 1.16 (1.07-1.25) |  |
| Severe food insecurity | 1.35 (1.24-1.48) |  |
| Sex |  |  |
| Male (reference) | 1.00 (1.00-1.00) |  |
| Female | 0.81 (0.78-0.84) |  |
| Age (ten years) | 0.94 (0.93-0.96) |  |
| Race-ethnicity |  |  |
| White (reference) | 1.00 (1.00-1.00) |  |
| Black | 0.58 (0.47-0.72) |  |
| Indigenous | 0.57 (0.50-0.64) |  |
| Others | 1.22 (1.12-1.32) |  |
| Not stated | 1.11 (0.95-1.30) |  |
| Immigrant status |  |  |
| Canadian-born (reference) | 1.00 (1.00-1.00) |  |
| Immigrant | 0.86 (0.81-0.91) |  |
| Not stated | 0.60 (0.42-0.86) |  |
| Household income (Canadian dollar) |  |  |
| Less than $20,000 (reference) | 1.00 (1.00-1.00) |  |
| $20,000 - 39,999 | 0.94 (0.87-1.02) |  |
| $40,000 - 59,999 | 0.88 (0.81-0.95) |  |
| $60,000 - 79,999 | 0.82 (0.75-0.89) |  |
| $80,000 or more | 0.78 (0.72-0.85) |  |
| Not stated | 0.96 (0.84-1.10) |  |
| Highest education in household |  |  |
| High school incomplete | 1.05 (0.97-1.13) |  |
| High school graduate (reference) | 1.00 (1.00-1.00) |  |
| Some college | 1.02 (0.93-1.12) |  |
| College degree | 0.99 (0.94-1.05) |  |
| Not stated | 1.10 (1.02-1.19) |  |
| Housing tenure |  |  |
| Renter (reference) | 1.00 (1.00-1.00) |  |
| Homeowner | 0.92 (0.87-0.97) |  |
| Not stated | 1.05 (0.75-1.46) |  |
| Household type |  |  |
| Couples with children (reference) | 1.00 (1.00-1.00) |  |
| Couples without children | 0.95 (0.89-1.00) |  |
| Lone parents | 1.07 (1.01-1.14) |  |
| Others | 1.00 (0.94-1.07) |  |
| Not stated | 1.14 (0.90-1.44) |  |
| Province of residence |  |  |
| Ontario (reference) | 1.00 (1.00-1.00) |  |
| Alberta | 1.26 (1.17-1.36) |  |
| Tobacco smoking status |  |  |
| Never smoked (reference) | 1.00 (1.00-1.00) |  |
| Former smoker | 1.04 (0.99-1.09) |  |
| Current smoker | 1.20 (1.15-1.25) |  |
| Not stated | 1.62 (1.12-2.33) |  |
| Past-year alcohol consumption |  |  |
| None | 1.10 (1.05-1.16) |  |
| Any up to once a week (reference) | 1.00 (1.00-1.00) |  |
| More than once a week | 0.90 (0.86-0.95) |  |
| Not stated | 1.27 (0.99-1.63) |  |
| CCHS cycle |  |  |
| Cycle 2005-2006 | 0.95 (0.88-1.03) |  |
| Cycle 2007-2008 | 0.93 (0.88-0.98) |  |
| Cycle 2009-2010 | 0.94 (0.88-1.00) |  |
| Cycle 2011-2012 | 0.95 (0.89-1.02) |  |
| Cycle 2013-2014 (reference) | 1.00 (1.00-1.00) |  |
| Cycle 2015-2016 | 1.01 (0.90-1.12) |  |
| Cycle 2017 | 0.94 (0.83-1.06) |  |
| Number of ED visit 13-24 months ago | 1.02 (1.01-1.02) |  |
| *Number of respondents* | *212,300* |  |
| Notes: ED = emergency department. FI = food insecurity. | |  |
|  |  |  |

| Table S5. Incidence rate ratio from Poisson models on past-year injury-related ED visits in overall sample and by sex and age subsamples | | | | |  |
| --- | --- | --- | --- | --- | --- |
|  | Food-secure | Marginal FI | Moderate FI | Severe FI |  |
| Injury-related ED visit, unadjusted (n=212,300) | reference | 1.19 (<0.0001) | 1.44 (<0.0001) | 1.87 (<0.0001) |  |
| Injury-related ED visit (n=212,300) | reference | 1.02 (0.72) | 1.16 (0.0002) | 1.35 (<0.0001) |  |
| Male (n=96,700) | reference | 0.998 (0.97) | 1.03 (0.60) | 1.37 (<0.0001) |  |
| Female (n=115,600) | reference | 1.06 (0.28) | 1.23 (0.0002) | 1.38 (<0.0001) |  |
| 12-17 years old (n=18,600) | reference | 0.96 (0.66) | 1.19 (0.021) | 1.25 (0.049) |  |
| 18-64 years old (n=140,200) | reference | 1.07 (0.15) | 1.16 (0.0006) | 1.43 (<0.0001) |  |
| 65+years old (n=53,500) | reference | 0.89 (0.57) | 1.29 (0.039) | 1.17 (0.40) |  |
| Notes: P-values are shown in parentheses after incidence rate ratios. ED = emergency department. FI = food insecurity. Unless specified otherwise, all Poisson models adjusted for sex, age, race-ethnicity, immigrant status, household income, income imputation status, highest education in household, housing tenure, household type, jurisdiction of residence, smoking status, past-year alcohol consumption, CCHS cycle, and number of ED visits in the year before. | | | | |  |
|  |  |  |  |  |  |

| Table S6. Sensitivity test on all-cause injury-related ED visits | | | | | |  |
| --- | --- | --- | --- | --- | --- | --- |
|  | Expanded sample (n=581,100) | Narrower outcome (n=212,300) | Weighted model (n=212,300) | Negative binomial (n=212,300) | Zero-inflated negative binomial (n=212,300) |  |
| Food-secure (reference) | 1.00 (1.00-1.00) | 1.00 (1.00-1.00) | 1.00 (1.00-1.00) | 1.00 (1.00-1.00) | 1.00 (1.00-1.00) |  |
| Marginal food insecurity | 1.09 (0.995-1.19) | 1.01 (0.93-1.10) | 1.03 (0.91-1.17) | 1.04 (0.96-1.12) | 1.03 (0.96-1.11) |  |
| Moderate food insecurity | 1.18 (1.09-1.27) | 1.15 (1.07-1.23) | 1.14 (1.02-1.27) | 1.16 (1.08-1.24) | 1.15 (1.08-1.23) |  |
| Severe food insecurity | 1.35 (1.24-1.47) | 1.33 (1.21-1.46) | 1.42 (1.21-1.68) | 1.31 (1.21-1.42) | 1.30 (1.20-1.41) |  |
| Notes: ED = emergency department. All models adjusted for sex, age, race-ethnicity, immigrant status, household income, income imputation status, highest education in household, housing tenure, household type, jurisdiction of residence, smoking status, past-year alcohol consumption, CCHS cycle, and number of ED visits in the year before. Expanded sample included jurisdiction-years with complete or partial ED records. Narrower outcome defined injury based on secondary causes only. Weighted model applied person weights. "Urban/rural residence status" was the predictor of the latent "zero injury" in the zero-inflated negative binomial model. | | | | | |  |
|  |  |  |  |  |  |  |

| Table S7. Poisson models on past-year ED visits due to cause-specific injury in overall sample | | | | |  |
| --- | --- | --- | --- | --- | --- |
|  |  |  |  |  |  |
|  | Food-secure | Marginal FI | Moderate FI | Severe FI |  |
| Intentional injuries | reference | 1.37 (0.14) | 1.54 (0.0055) | 1.81 (0.0005) |  |
| Self-harm | reference | 1.61 (0.21) | 1.51 (0.17) | 1.87 (0.039) |  |
| Violence | reference | 1.27 (0.31) | 1.56 (0.014) | 1.79 (0.0047) |  |
| Non-intentional injuries | reference | 1.01 (0.88) | 1.14 (0.0007) | 1.34 (<0.0001) |  |
| Fall | reference | 1.07 (0.36) | 1.22 (0.0010) | 1.43 (<0.0001) |  |
| On same level | reference | 1.10 (0.35) | 1.11 (0.21) | 1.15 (0.22) |  |
| On stairs | reference | 1.16 (0.43) | 1.71 (0.0001) | 2.24 (<0.0001) |  |
| Others | reference | 1.00 (0.98) | 1.18 (0.07) | 1.49 (0.0003) |  |
| Medical complication | reference | 0.83 (0.38) | 1.12 (0.55) | 1.39 (0.017) |  |
| Surgical | reference | 0.84 (0.46) | 1.05 (0.81) | 1.32 (0.10) |  |
| Non-surgical | reference | 0.83 (0.55) | 1.17 (0.64) | 1.49 (0.055) |  |
| Struck-by | reference | 1.06 (0.60) | 1.20 (0.034) | 1.43 (0.015) |  |
| Falling objects | reference | 1.15 (0.56) | 1.20 (0.41) | 1.19 (0.54) |  |
| In sports | reference | 0.97 (0.87) | 1.02 (0.90) | 0.89 (0.66) |  |
| In non-sports | reference | 1.14 (0.37) | 1.40 (0.0042) | 1.83 (0.0015) |  |
| Transport | reference | 1.03 (0.80) | 1.05 (0.65) | 1.29 (0.11) |  |
| Pedestrian or cyclist | reference | 0.87 (0.59) | 1.15 (0.55) | 1.00 (0.998) |  |
| Motor vehicle | reference | 1.06 (0.68) | 0.95 (0.68) | 1.38 (0.08) |  |
| Overexertion | reference | 0.94 (0.57) | 1.25 (0.017) | 1.31 (0.024) |  |
| Animal bite or sting | reference | 1.27 (0.17) | 1.20 (0.28) | 1.60 (0.019) |  |
| Skin piercing | reference | 1.05 (0.83) | 0.99 (0.97) | 1.80 (0.0034) |  |
| Poisoning | reference | 0.62 (0.61) | 1.16 (0.80) | 1.65 (0.029) |  |
| Other non-intentional injuries | reference | 1.00 (0.98) | 1.09 (0.20) | 1.32 (0.0001) |  |
| Notes: P-values are shown in parentheses after incidence rate ratios. ED = emergency department. FI = food insecurity. The "adjusted" Poisson models controlled for sex, age, race-ethnicity, immigrant status, household income, income imputation status, highest education in household, housing tenure, household type, jurisdiction of residence, smoking status, past-year alcohol consumption, CCHS cycle, and number of ED visits in the year before. | | | | |  |
|  |  |  |  |  |  |


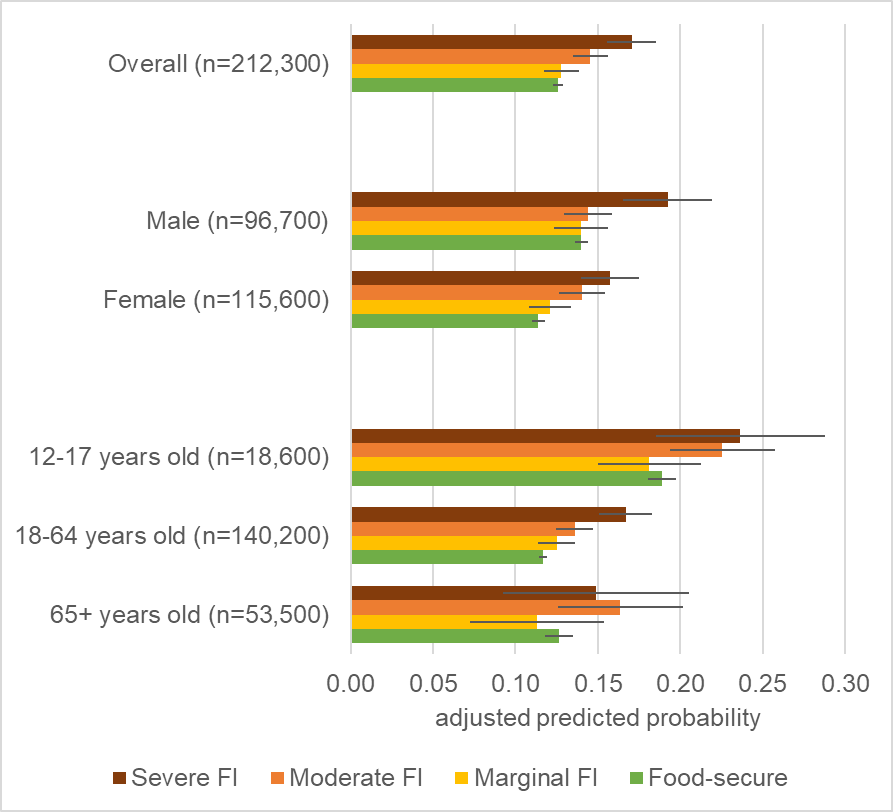


Figure S1. Adjusted predicted probability of injury by food insecurity status: overall sample and by sex and age groups.

Black lines represent 95% confidence intervals. Average probabilities were estimated through marginal standardization based on the adjusted Poisson models.


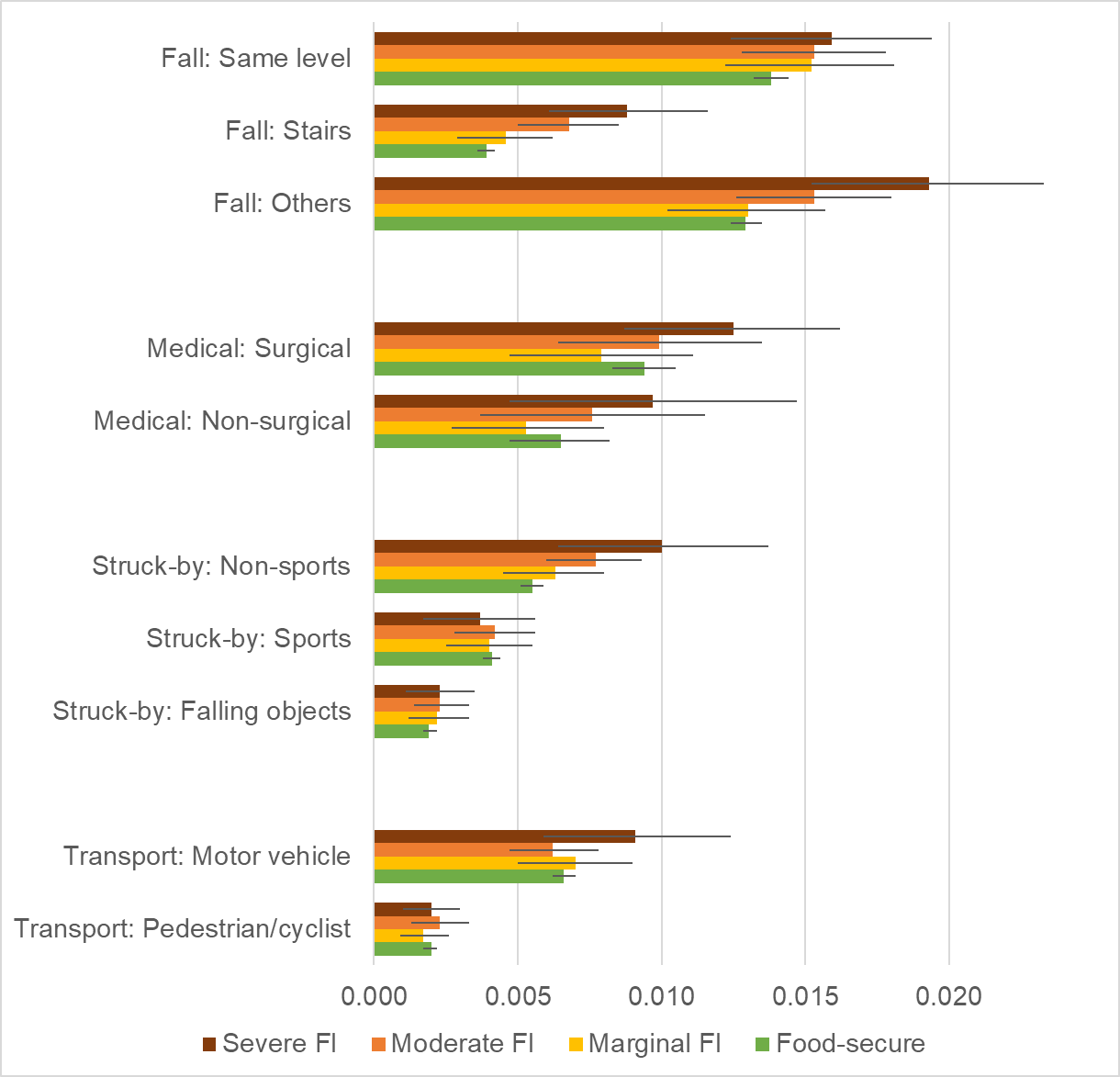


Figure S2. Adjusted predicted probability of specific non-intentional injury by food insecurity status: overall sample.

Black lines represent 95% confidence intervals. Average probabilities were estimated through marginal standardization based on the adjusted Poisson models.
